# Mechanical Behavior of Octopus Egg Tethers Composed of Topologically Constrained, Tandemly Repeated EGF Domains

**DOI:** 10.1021/acs.biomac.3c00088

**Published:** 2023-06-09

**Authors:** William R. Wonderly, Daniel G. DeMartini, Saeed Najafi, Marcela Areyano, Joan-Emma Shea, J. Herbert Waite

**Affiliations:** † Department of Chemistry & Biochemistry, University of California Santa Barbara, Santa Barbara, California 93106, United States; ‡ Department of Molecular, Cell, and Developmental Biology, University of California Santa Barbara, Santa Barbara, California 93106, United States; § Materials Research Laboratory, University of California Santa Barbara, Santa Barbara, California 93106, United States; ∥ Department of Mechanical Engineering, University of California Santa Barbara, Santa Barbara, California 93106, United States; ⊥ Department of Physics, University of California Santa Barbara, Santa Barbara, California 93106, United States

## Abstract

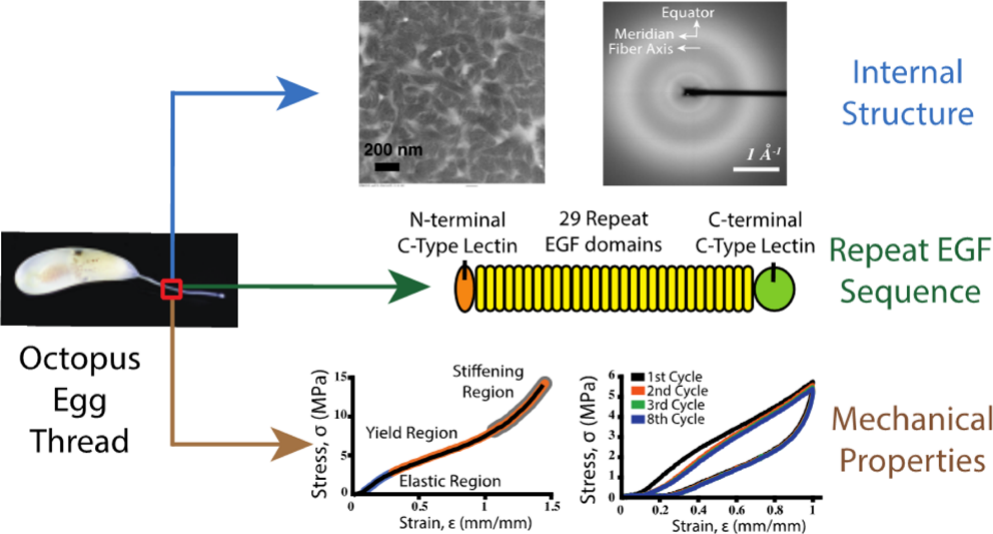

Whether and how intramolecular crosslinks in polymeric materials contribute to mechanical properties is debated in both experimental and theoretical arenas. The tethering threads of *Octopus bimaculoides* egg cases provide a rare window to investigate this question in a biomaterial. The only detectable component of the load-bearing fibers in octopus threads is a 135 kDa protein, *octovafibrin*, comprising 29 tandem repeats of epidermal growth factor (EGF) each of which contains 3 intramolecular disulfide linkages. The N- and C-terminal C-type lectins mediate linear end-to-end octovafibrin self-assembly. Mechanical testing of threads shows that the regularly spaced disulfide linkages result in improved stiffness, toughness, and energy dissipation. In response to applied loads, molecular dynamics and X-ray scattering show that EGF-like domains deform by recruiting two hidden length β-sheet structures nested between the disulfides. The results of this study further the understanding of intramolecular crosslinking in polymers and provide a foundation for the mechanical contributions of EGF domains to the extracellular matrix.

## Introduction

Intermolecular crosslink density determines most of the critical mechanical properties of polymeric materials.^1^ Contrasted with this, the long-standing view of *intra*molecular crosslinks has been that they contribute minimally to the mechanical properties of synthetic systems,^2^ but this is increasingly being called into question. One study on the bulk mechanical properties of a single-chain nanoparticle (SCNP), for example, has found that introducing intramolecular crosslinks can increase the stiffness and extensibility.^3^ Given this, further exploring mechanisms by which intramolecular crosslinks contribute to and influence load bearing in polymers shows promise, notwithstanding the limitations of synthetic SCNPs including scalability, crosslinking selectivity, domain size, and number of available 3D architectures.^4^

Biological intramolecular crosslinks, by contrast, are widespread, precisely positioned, and often recruited into load bearing with the potential to provide insights without synthetic limitations. For example, mechanical studies on different immunoglobulin (Ig) modules from titin, which contribute to the extensibility and energy dissipation of titin, have shown that specific intramolecular hydrogen bonds break during domain unfolding in response to deformation.^5,6^ Additionally, intramolecular interactions in elastin^7^ and spider silks^8^ are present as covalent bonds and nanocrystalline β sheets, respectively. A further aspect of intramolecular crosslinks is that they introduce topological constraints, thereby reducing the conformational landscape that a protein can assume. Proteins that possess non-trivial geometrical motifs in their native state (*i.e.*, polypeptides that are intertwined, or knotted, or have covalently stabilized loops) that cause them to be self-constrained often yield unexpected mechanical effects. Numerous computational and experimental studies have investigated how topological constraints affect the mechanical response of a protein.^9−18^ However, these studies focus on the response of individual proteins rather than bulk material properties. Therefore, systems in which correlations between crosslinking, complex topology, and bulk mechanical properties can be investigated are highly desirable. Biomolecular materials that contain proteins with epidermal growth factor (EGF) repeats are excellent candidates.

**Figure 1:**
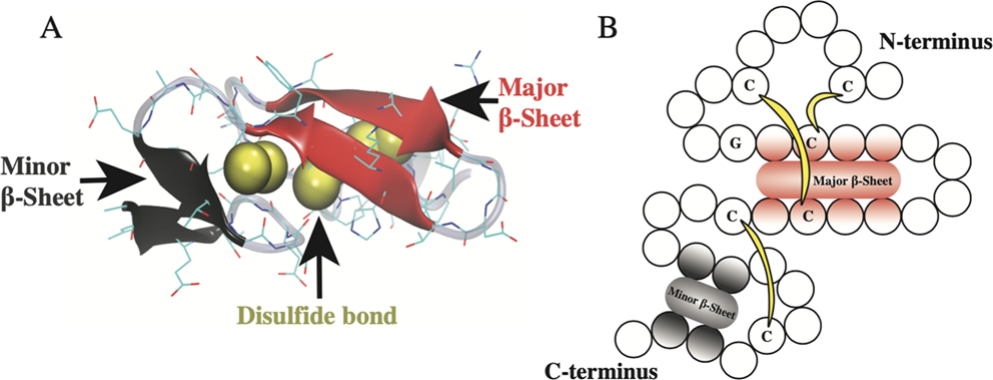
EGF domains. (A) Molecular model of an EGF domain that contains a major β-sheet (red), minor β-sheet (black), and three disulfide bonds (yellow spheres). (B) Cartoon representation of the EGF topology with certain conserved residues indicated.

EGF-like domains (Figure 1) are short polypeptide sequences (∼30–40 amino acids long) with 6 conserved cysteines that form three intramolecular disulfide (DS) bonds. DS bonds create three constrained loops in the native structure by canonical pairing of six conserved Cys residues (Figure 1B).^19^ EGF domains are thus self-constrained motifs that, despite being commonly found in the extracellular matrix (ECM), possess largely unknown mechanical behaviors.^20^

EGF-like domains participate in a number of critical load bearing, cellular recognition, signaling, and mitogenic and growth functions.^21^ The most notable load-bearing EGF-rich protein, fibrillin, contains as many as 47 tandem EGF repeats and 141 intramolecular crosslinks.^22^ Fibrillin thus appears ideal for investigating the mechanical response of single-molecule EGF-like domains under tension, but several confounding issues arise in testing these bulk materials. Fibrillin is typically found in the presence of elastin to such an extent that fibrillin is suggested to provide a scaffold for elastin deposition;^23^ the mechanical response of the EGF-like domains is thus obscured by the association of other proteins including elastin. Additionally, fibrillin features a complex “pseudo-beaded string” ultrastructure, whose molecular nature is disputed,^24−26^ and the mechanical response of fibrillin has largely been explained in terms of the ultrastructure and global deformation rather than the segmental rearrangements of the individual EGF motifs. Indeed, investigations of other proteins with EGF-like domains have largely ignored their mechanical contribution.^27,28^ Proteins with tandemly repeated sequences such as EGF often result in the formation of solenoid supercoiling that ranges from linear to circular.^29^

Inspired by previous studies on protective egg casings,^30,31^ we discovered that the thread tethers of *Octopus bimaculoides* egg cases are ideal model systems for assessing the mechanical contributions of EGF domains under tension. The threads are robust and persist through a 1–3 month-long brooding period.^32^ More to the point, a single protein, *octovafibrin* (octopus ovarian fibrous protein), appears to comprise both the threads and egg cases. *Octovafibrin* consists of 29 tandemly repeated EGF units, terminated at both N- and C-ends by lectin binding domains. Because each egg case tapers into a 1–3 cm-long thread at the proximal end, the threads can be easily separated and are ideally suited for testing both static/cyclic mechanical and optical properties under tension. We employed coarse-grained (CG) molecular dynamics (MD) simulations to characterize the influence of the topological constraints of EGF-like motifs, created by the intramolecular DS bonds, on the mechanical response of the octopus egg threads. To understand the molecular mechanism and the subsequent local structural unfolding by which EGF-like domains respond to mechanical loading, a combination of wide-angle X-ray scattering (WAXS) and molecular modeling techniques was used. Finally, we investigated the effects of intramolecular disulfide bonds by analyzing the mechanical response of threads after treatment with a reducing agent.

## Materials and Methods

### Egg Thread Collection and Microscopy

Adult *O. bimaculoides* specimens were collected near Santa Barbara, CA, and kept in an open seawater system at the University of California Santa Barbara. Octopuses were held for several weeks and monitored for egg laying. Once the females laid eggs, the egg bundles were collected and freshly separated under a dissecting microscope to obtain tens of milligrams of isolated egg threads. The egg threads were then rinsed in filtered (0.2 μm) seawater before experiments were conducted.

### Electron Microscopy

The egg threads were prepared for scanning electron microscopy (SEM) by fixing in 1% formaldehyde in marine phosphate-buffered saline (0.05 M sodium phosphate, 0.45 M NaCl, pH = 7.4), both in the relaxed state and under 100% strain, for 4 h at 4 °C. Next, the egg threads were rinsed with fresh water and then transferred to 100% ethanol through a graded series of solutions. The egg threads were then transferred into 100% hexamethyldisilazane (HMDS) through a series of HMDS/ethanol solutions. Finally, the egg threads were dried directly on SEM stubs covered with carbon tape and silicon wafer, coated with a thin film of gold (JFC-16000 sputter coater, JEOL), and imaged using a FEI Nova Nano 650 FEG SEM operating at 3 keV.

Egg threads were prepared for transmission electron microscopy (TEM) by fixing in 2% formaldehyde and 2.5% glutaraldehyde in fixation buffer (200 mM sodium cacodylate, 300 mM NaCl, pH 7.2) for 2 h on ice. The samples were washed three times (10 min each) in degassed fixation buffer and then post-fixed in 2% osmium tetroxide in degassed fixation buffer for 2 h. Following this step, the samples were washed four times (10 min each) at room temperature with degassed deionized water and then dehydrated through a graded series of ethanol washes. The sample was then transferred from ethanol into 100% propylene oxide in a graded solvent series. The samples were submerged in resin diluted in propylene oxide to infiltrate them with epoxy resin (Embed812, Electron Microscopy Sciences, Hatfield, USA) as follows: 33% (2 h), 66% (16 h), and 100% (4 h). Finally, the samples were placed in silicon molds and cured at 60 °C for 24 h. Thin sections (60–80 nm) for TEM and semi-thin sections (500 nm) for bright-field light microscopy were cut on an EM UC6 Ultramicrotome (Leica Biosystems, Wetzlar, Germany). TEM sections were mounted on copper grids (CF200, Electron Microscopy Sciences, Washington D.C., USA) and post-stained with drops of uranyl acetate and lead citrate following standard protocols.^33^

### SDS–PAGE

Fresh egg threads were rinsed in deionized water and placed directly into sodium dodecyl sulfate-polyacrylamide gel electrophoresis (SDS-PAGE) sample buffer (50 mM Tris HCl, 10% v/v glycerol, 2% w/v SDS, and 0.1% w/v bromophenol blue, pH 6.8) with and without 100 mM β-mercaptoethanol (β-ME), and with and without heating to 95 °C, for 5 min. Samples were resolved on 10% Tris-glycine acrylamide protein gels, at 125 V, for 50 min. The protein molecular weight was estimated based on a standard protein ladder.

The major protein of the egg thread, *octovafibrin*, was resolved by SDS-PAGE and the band extracted for an in-gel trypsin digest and prepared for and analyzed by LC/MS/MS following standard protocols described by the University of California San Francisco Mass Spectrometry Facility.^34,35^ Several protein fragments were *de novo* sequenced based on the fragmentation patterns produced by collision-induced decomposition.

### Amino Acid Analysis

Amino acid analysis was performed on a Hitachi L-8900 ninhydrin-based amino acid analyzer. Fresh egg threads (1 day old) and aged egg threads (100 days old) were analyzed for quantitative protein and amino acid contents. The putative 135 kDa gel band was also analyzed for comparison. A freshly run gel was electro-transferred to a poly(vinylidene difluoride) membrane with a transblot apparatus (Bio-Rad) in transfer buffer (25 mM Tris, 192 mM glycine, and 20% v/v methanol) at 25 V for 15 min. The membrane was cut out around the 135 kDa band. All protein samples for amino acid analysis were hydrolyzed in 6 M HCl under vacuum at 110 °C for 24 h, rinsed multiple times with water and methanol, reconstituted in 0.02 M HCl, and loaded onto the analyzer.

### Inductively Coupled Plasma-Atomic Emission Spectroscopy

Inductively coupled plasma–atomic emission spectroscopy was performed on a Thermo iCAP 6300 with a sample uptake rate of 1.5 mL/min. Egg threads were prepared by hydrolysis in 5% nitric acid and diluted to 1% before analysis. Calcium concentrations in the egg thread were the same as ambient seawater.

### Mechanical Tensile Testing

Tensile loading and cyclic loading tests were performed on a tabletop tensile tester (Bionix 200 universal testing machine, MTS, Eden Prairie, MN), at a nominal strain rate of 1.0 min^–1^, using a 10 N load cell and a built-in optical encoder to measure the load and displacement. Motor command was performed with the built-in MTS TestSuite software (MTS, Eden Prairie, MN). All mechanical tests were performed with the threads fully submerged in filtered seawater inside an environmental chamber (Bionix 200 universal testing machine, MTS, Eden Prairie, MN). Threads were clamped with stainless steel grips, and those under tensile loading experiments were pulled to failure. Threads undergoing cyclic loading were pulled to various strains and returned to zero strain. Threads that broke at the clamp interface were not recorded. Mechanical tests were run under multiple conditions and configurations. Stress relaxation experiments were conducted in the same manner, and samples were adhered to tabs and pulled on at a strain rate of 100 min^–1^. The strain was held constant for 10 min, and stress was recorded over this time. To study the effect of reducing DS linkages, threads were pre-treated in artificial seawater supplemented with a range of dithiothreitol (DTT) concentrations (0.001, 0.01, 0.1, 1.0, and 10 mM) for 1 h prior to testing. All mechanical data were processed using MATLAB (MathWorks, Natick, MA).

### Transparency Observations

The transparency of each egg thread changed during cyclic loading. Photographs of the thread were taken at different strains using a Canon Rebel SLR during successive pulling cycles as described above. The change in contrast relative to the background (Weber contrast) was used to represent change in transparency. The integrated intensity of the threads and background were analyzed using ImageJ (NCBI).

### Wide-Angle X-ray Scattering

Transmission WAXS experiments were performed at the University of California Santa Barbara Materials Research Lab X-ray diffraction facility. Diffraction patterns were acquired using a custom-built X-ray diffractometer equipped with a 50 μm microfocus 1.54 Å Cu X-ray source (Genix from XENOCS SA, France) and an EIGER R 1M solid-state detector (Dectris, Switzerland) at a sample-to-detector distance of 157 mm.

Threads were pulled to 70% strain and held at this strain while drying using a home-built strain gauge. After drying, the threads remain at 70% strain. Strained threads (10 in total) were bundled together and affixed to the sample holder. Measurements of the unstrained state were performed on 10 pristine threads bundled together. Prior to measurement, the threads were aligned and dried.

### Molecular Modeling

A CG representation of the *O. bimaculoides* thread topology and structure was obtained by employing an elastic polymer model relative to the native thread state. The native structure of the thread was constructed by connecting 29 aligned EGF-like units. The EGF-like unit was extracted from the crystal structure of the *neurogenic locus notch homolog* protein.^36^ The CG model (Supporting Figure S1) was based on the spatial organization of the C_α_ atoms of the polypeptide. Each amino acid of the thread is represented by a simple monomeric unit with unit length σ = 3.8 Å. The monomers are covalently bonded to the first adjacent monomers along the polypeptide chain through a finite-extensible-nonlinear-elastic potential. The monomers are subject to two short-range interactions: (i) the steric hindrance interaction that prevents the chain from self-crossing, featured by the Weeks–Chandler–Andersen potential and (ii) the disulfide bond interaction that is modeled by a strong attractive Gaussian potential. The elastic polymer model of the thread was then provided with the bending and torsion potentials that are exerted to the triplets and quadruplets of successive monomers, respectively, whose reference angles are parametrized based on the thread native structure. Further details about the model and MD simulations protocol are described in the Supporting Information.

## Results

### Specimen Description

**Figure 2:**
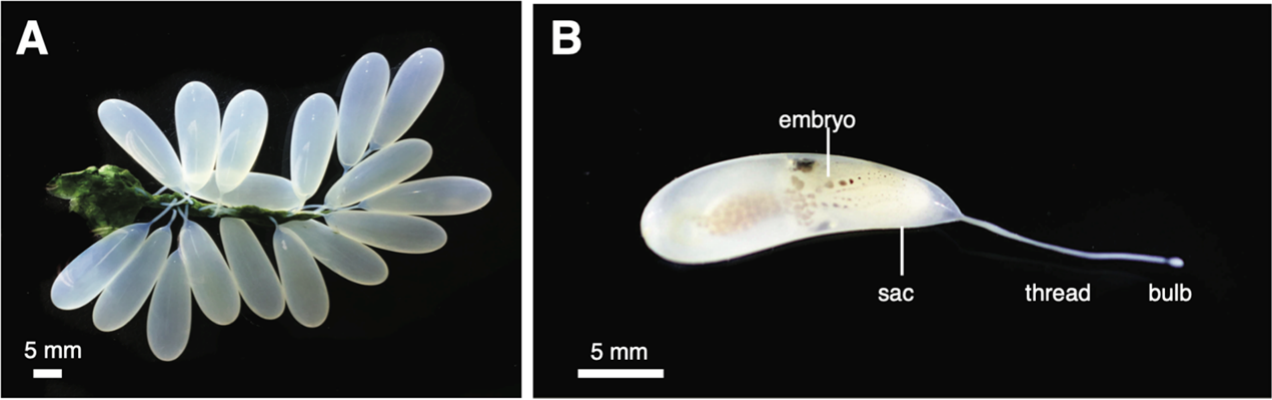
Dissecting microscopic image of *O. bimaculoides* eggs. (A) Multiple eggs are attached to a central, green, and cement stalk. (B) Individual eggs are surrounded by a white sac that tapers into a thread and terminates in a small bulb.

As deposited by the California two-spot octopus (*O. bimaculoides*), multiple eggs were clustered together and secured to a substratum by a dark green cement (Figure 2A).

Individual eggs consisted of a roughly pear-shaped embryo encased in a translucent white sheath. At the base of the eggs, the sheath tapered into an opaque white thread that is typically 6–15 mm long and 0.4 mm in diameter and terminates as a bulb (Figure 2B).

**Figure 3:**
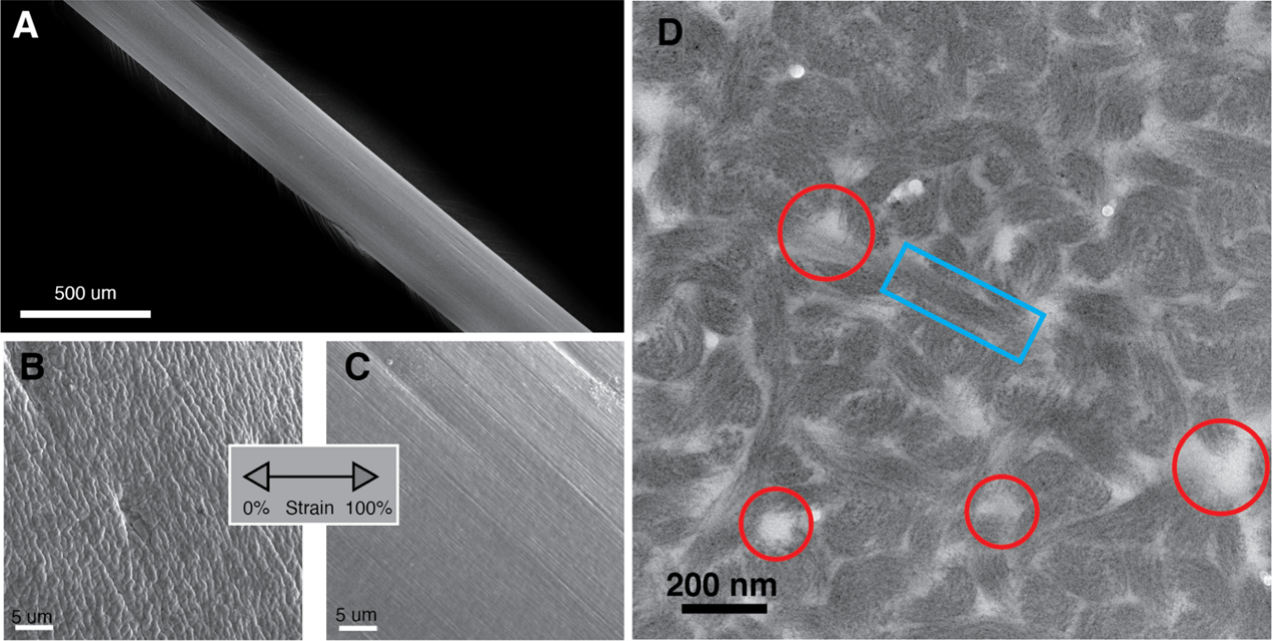
Electron microscopy images of octopus threads. (A) Low magnification SEM image of a single, unstrained, octopus thread. (B) SEM image of the surface of an unstrained octopus thread showing a coarse surface. (C) SEM image of the surface of an octopus thread pulled to 100% strain that shows ridges aligned with the long axis of the thread. (D) TEM image of a horizontal cross-section of an unstrained octopus thread. One example of a fibrous bundle is highlighted with a light blue rectangle, whereas red circles indicate voids that likely scatter light.

Electron microscopy studies revealed the micron and submicron structure in the egg threads. SEM showed that the threads were approximately 400 μm in diameter (Figure 3A). In a relaxed state, the surface of the egg thread was wrinkled and amorphous (Figure 3B). Egg threads held at 100% strain, however, showed a striated surface with ridges parallel to the axial loading direction (Figure 3C).

TEM images of transverse sections (Figure 3D) showed that 200 nm-thick fibrous bundles make up the bulk of the egg thread (blue box), with small voids scattered throughout the internal structure (red circles). Within the bundles, fibers were aligned in the same direction; however, globally, the bundles were isotropic.

### Biochemical Characterization

**Figure 4:**
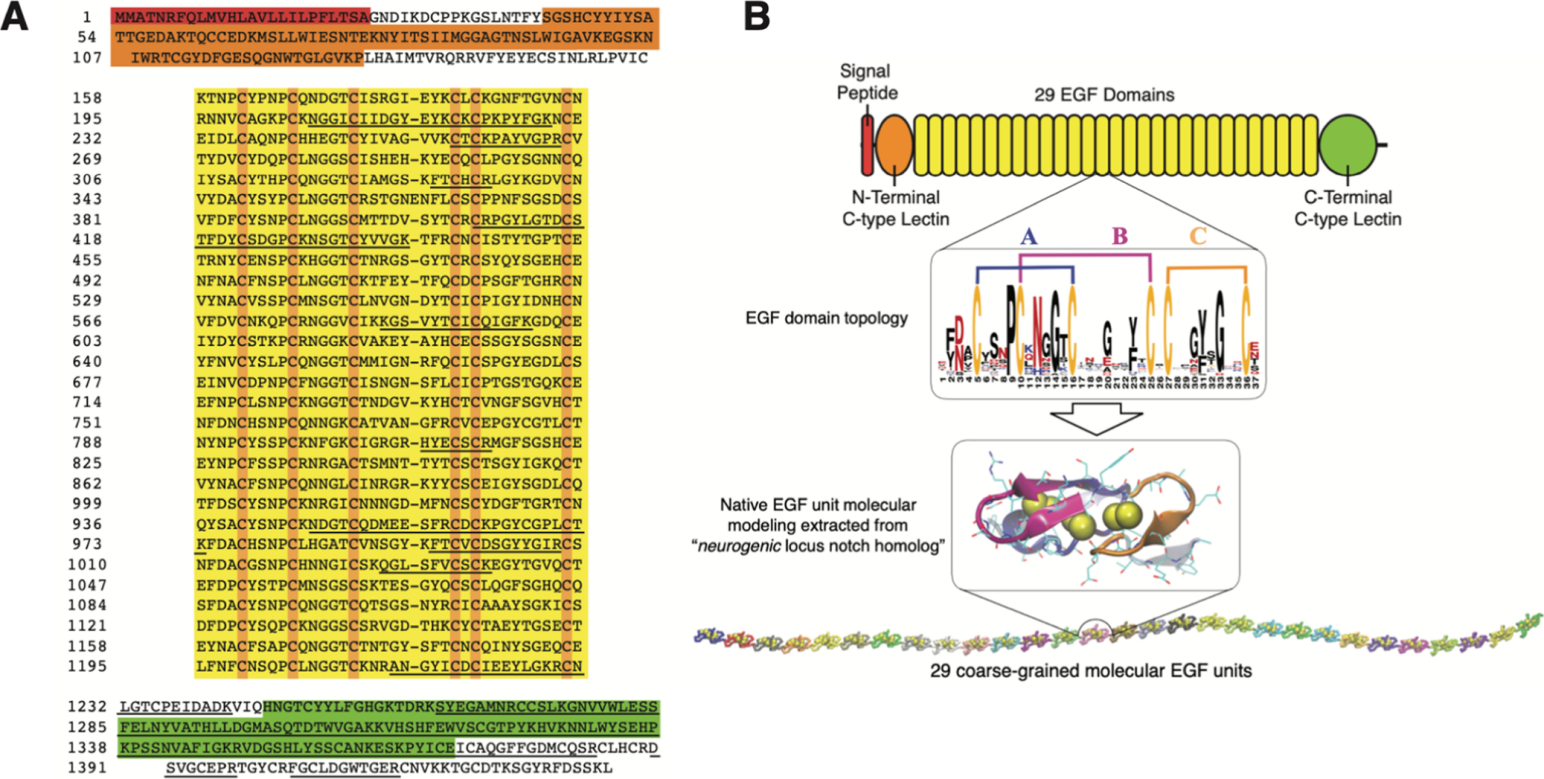
Sequence and structure of *octovafibrin*. (A) The predicted sequence of *octovafibrin* shows a secretory processing signal peptide (red), an N-terminal C-type lectin binding domain (orange), 29 EGF domain repeats (yellow, with an emphasis on conserved cysteines), and a C-terminal C-type lectin binding domain (green). Sequences verified by MS/MS are underlined. (B) Cartoon representation of *octovafibrin*. The zoom of EGF structure is depicted with disulfide bonds blue (DS-A, C6–C16), pink (DS-B, C27–C36), and orange (DS-C, C27–C36). The template-based EGF molecular model is also shown with disulfide bonds depicted as yellow spheres. We linearly link 29 identical EGF units to mimic the central EGF sequence of *octovafibrin*.

Homogenization of the egg threads in Tris buffer at either room temperature or 95 °C yielded no discernable protein, as analyzed by SDS-PAGE (Supporting Figure S2A, lanes 3 and 5). The addition of β-ME, however, readily solubilized the threads to generate a single major protein band (Supporting Figure S2A, lane 4). Addition of heat increased the extraction efficiency (Supporting Figure S2A, lane 2). The apparent molecular weight of *octovafibrin* was determined to be ∼135 kDa from its mobility during SDS-PAGE (Supporting Figure S2B). Partial sequences of *octovafibrin* were obtained by in-gel trypsin digestion, followed by MS/MS (Table S1), which were then used to find the full-length sequence from the *O. bimaculoides* genome^37^ (NCBI accession no. XP_014771937.1, Figure 4A). A C-type lectin binding domain, followed by 29 consecutive EGF-like domains, ending in a C-terminal C-type lectin binding domain (Figure 4B) characterizes the full *octovafibrin* sequence. The consensus sequence of the EGF-like domains shows completely conserved cysteines at the 5th, 10th, 16th, 25th, 27th, and 36th positions. A query of this sequence using PROSITE^38^ indicates that none of the EGF domains are calcium binding.

However, the large number of D/N residues in the 3rd position and Y/F residues in the 23rd position are partly consistent with the consensus calcium binding EGF motif.^39^ A template-based model of a representative EGF domain is also presented to illustrate the major and minor β-sheets along with the assembly of 29 consecutive EGF domains used for CG MD simulations (Figure 4B). The similarity between the amino acid compositions of whole threads and the single major 135 kDa protein band extracted from the protein gel indicate that threads are primarily composed of *octovafibrin* assemblies (Supporting Figure S3B). The amino acid content does show some discrepancy in the cysteine contents between the threads, protein extract, and predicted sequence; however, this was to be expected given that only cystine is not destroyed during protein hydrolysis with 6 N HCl and that the cystine levels in the thread are likely trebled over those in purified octovafibrin by intermolecular crosslinking in the N-/C-termini.^40^

### Mechanical Property Characterization

**Figure 5:**
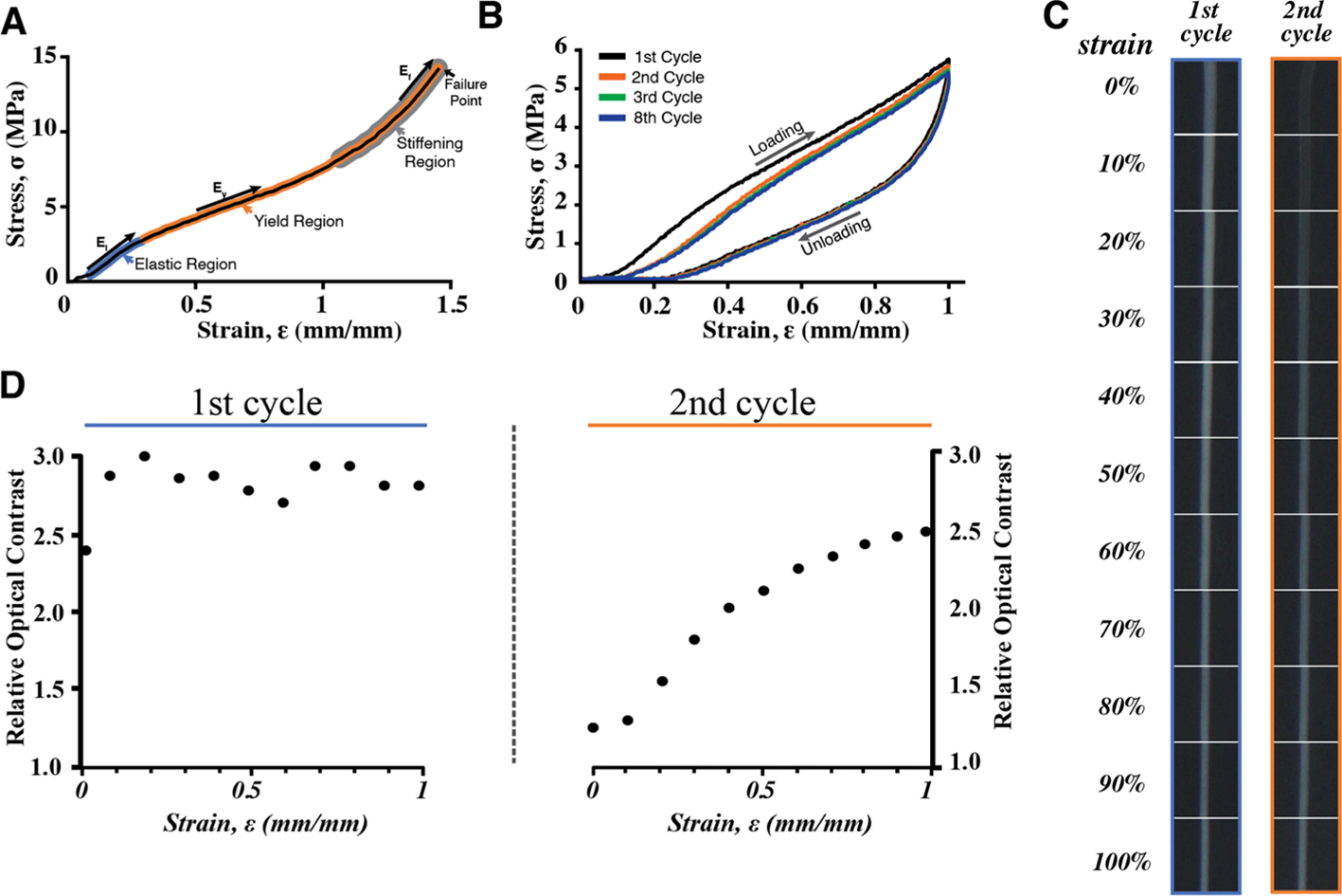
Mechanics and optical changes of threads under tensile loading. (A) Typical stress–strain curve (strain rate = 1.0 min^–1^) of an octopus thread shows an initial elastic region (blue), followed by a low modulus softening region (orange) and ending with a stiffening region (gray) prior to failure. (B) Typical stress behavior of an octopus thread under repeated cyclic load. The 1st (red), 2nd (orange), 3rd (green), and 8th (blue) cycles are shown. (C) The left column shows dissecting microscopic images of pristine threads at different strain values during an initial load cycle, while the right column shows images of threads during the second load cycle. (D) Plots of relative optical (Weber) contrast as a function of strain during the first (left plot) and second (right plot) loading cycles.

When strained to failure, octopus egg threads exhibited three distinct regimes: (i) an initial elastic region to about 30% with a modulus of about 8.5 ± 1.0 MPa, (ii) a softening region with a decrease in modulus (4.6 ± 0.5 MPa) to strains of about 100%, and (iii) a stiffening region (*E* = 11.6 ± 3.7 MPa) that spanned to nearly 140% strain whereupon the thread failed (Figure 5A). When treating threads with EGTA, which removes calcium, we observed no difference in mechanical behavior (Figure S4). During repeated cyclic loading of a single egg thread, modulus decreased slightly after the first cycle (Figure 5B).

The average toughness and hysteresis values of threads changed when cycled 8 times between 100 and 0% strain (Supporting Table S3). Pristine threads achieved an average toughness of 2.48 MJ/m^3^ with 53% hysteresis. During the second cycle, threads had a lower toughness of 2.12 MJ/m^3^ and 48% hysteresis. These values decreased slightly over subsequent cycles. The loading curve showed damage and reduced hysteresis after the first cycle and did not undergo further change during subsequent cycles (2–8). The unloading behavior was the same regardless of cycle number. The strain history, reduced modulus, and softening were permanent as the thread properties remained unchanged after 24 h of rest (Supporting Figure S5).

Optical changes in the egg threads were also observed (Figure 5C,D). Pristine egg threads were white and maintained a constant optical contrast through their first load cycle. Upon unloading, the egg threads became transparent and exhibited little optical contrast. During the second (and all subsequent) load cycles, the threads regained optical contrast upon straining and became transparent upon unloading.

The cyclic loading to increasing strains on a single thread, for a total of 12 cycles, is shown in Supporting Figure S6. The first cycle ended at 10% strain, and the endpoint of each subsequent cycle was increased by 10% strain. A decrease in the modulus was observed in each subsequent cycle and thus leading to different loading paths (Supporting Figure S6A). An analogous chart constructed of cyclic loading curves of pristine threads pulled to different strain values is presented to show loading/unloading trajectories (Supporting Figure S6B). To probe the viscoelastic properties of octopus threads, we performed a stress relaxation experiment (Supporting Figure S6C). We found that when a thread is held at 70% strain, it relaxes to nearly 60% of the maximum stress achieved within 100 s. Attempts to model this behavior with a simple exponential decay function or Maxwell model were unsuccessful and as such we do not report a relaxation time. Further studies may try using a Prony series to model this behavior.^41^

**Figure 6:**
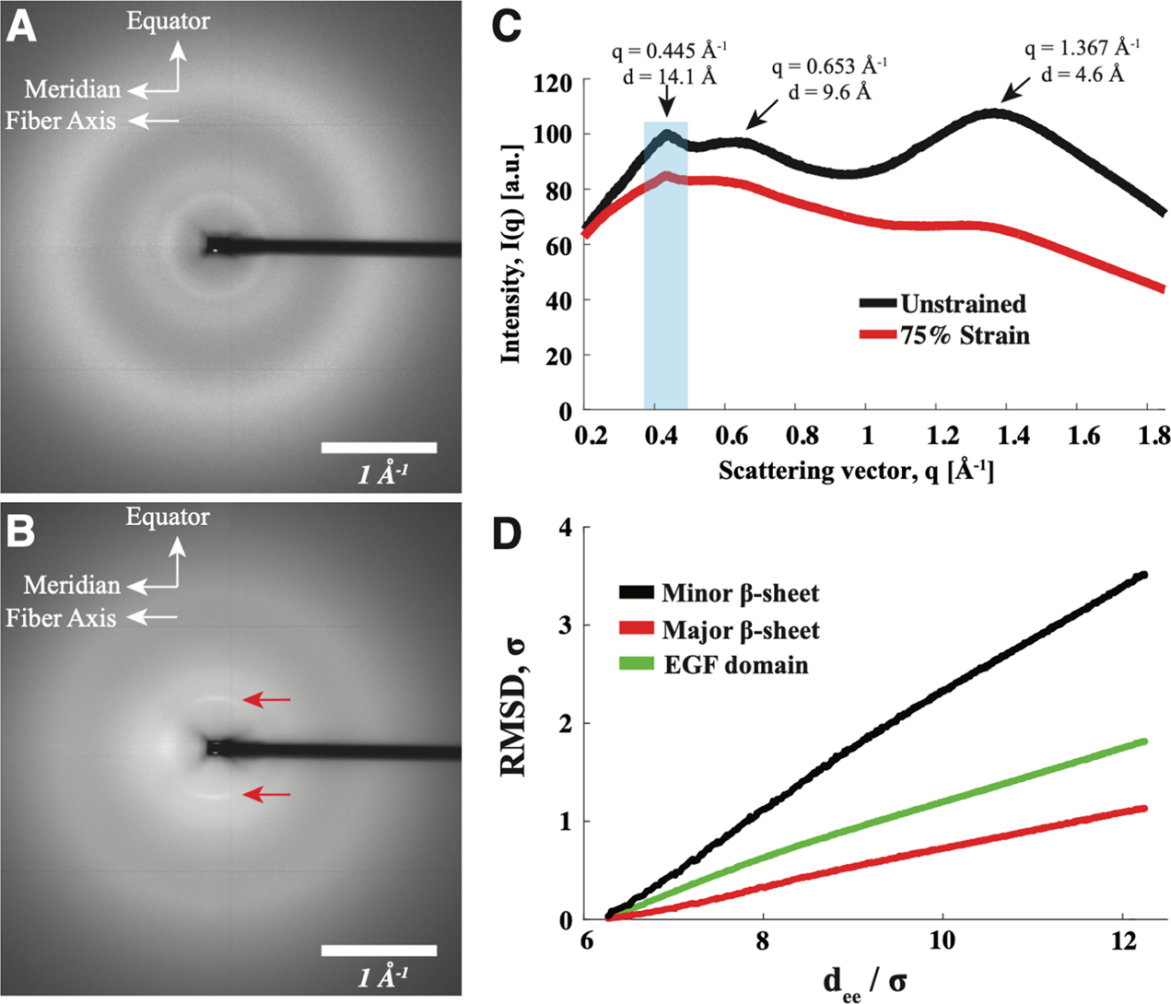
WAXS analysis of pristine octopus threads and threads held at 70% strain. (A) 2D WAXS pattern of dry, unstrained, octopus threads. (B) 2D WAXS pattern of dried threads at 70% strain. (C) Integrated 1D radial intensity profiles of the unstrained threads (black) and strained threads (red). The shaded blue box indicates the area to generate the 1D azimuthal profile of the peak at *q* = 0.445 Å^–1^ (see Supporting Figure S7). (D) RMSD calculation of a strained EGF domain from CG-MD simulation showing the unfolding of the minor β-sheet (black), major β-sheet (red), and full EGF domain (green).

WAXS was used to probe the strain-related changes in a secondary structure. The 2D WAXS patterns (Figure 6A,B) show that the threads are largely isotropic, with some anisotropy arising at low *q* for strained threads (red arrows). The 1D radial profiles of unstrained and strained threads (Figure 6C) identify three features at *q* = 0.445, 0.653, and 1.367 Å^–1^. We further observed that, when strained, the feature at 1.367 Å^–1^ decreased in intensity relative to the other features. The azimuthal intensity distribution (Supporting Figure S7) showed that, when strained, the feature at 0.445 Å^–1^ aligned in the equatorial direction.

To understand how EGF domains are deformed, we simulated strain in an EGF domain (Figure 6D). The root-mean-square deviation (RMSD) value for the minor β-sheet increased the most, indicating that it unfolded readily (black). The major β-sheet experiences much less disruption (red) due to the fact that it is strongly constrained by the disulfide bonds. As a result, the whole EGF domain partially unfolded (green). We also modeled the result of a fully reduced EGF domain (Supporting Figure S8) and found that this resulted in rapid denaturation of all structures.

**Figure 7:**
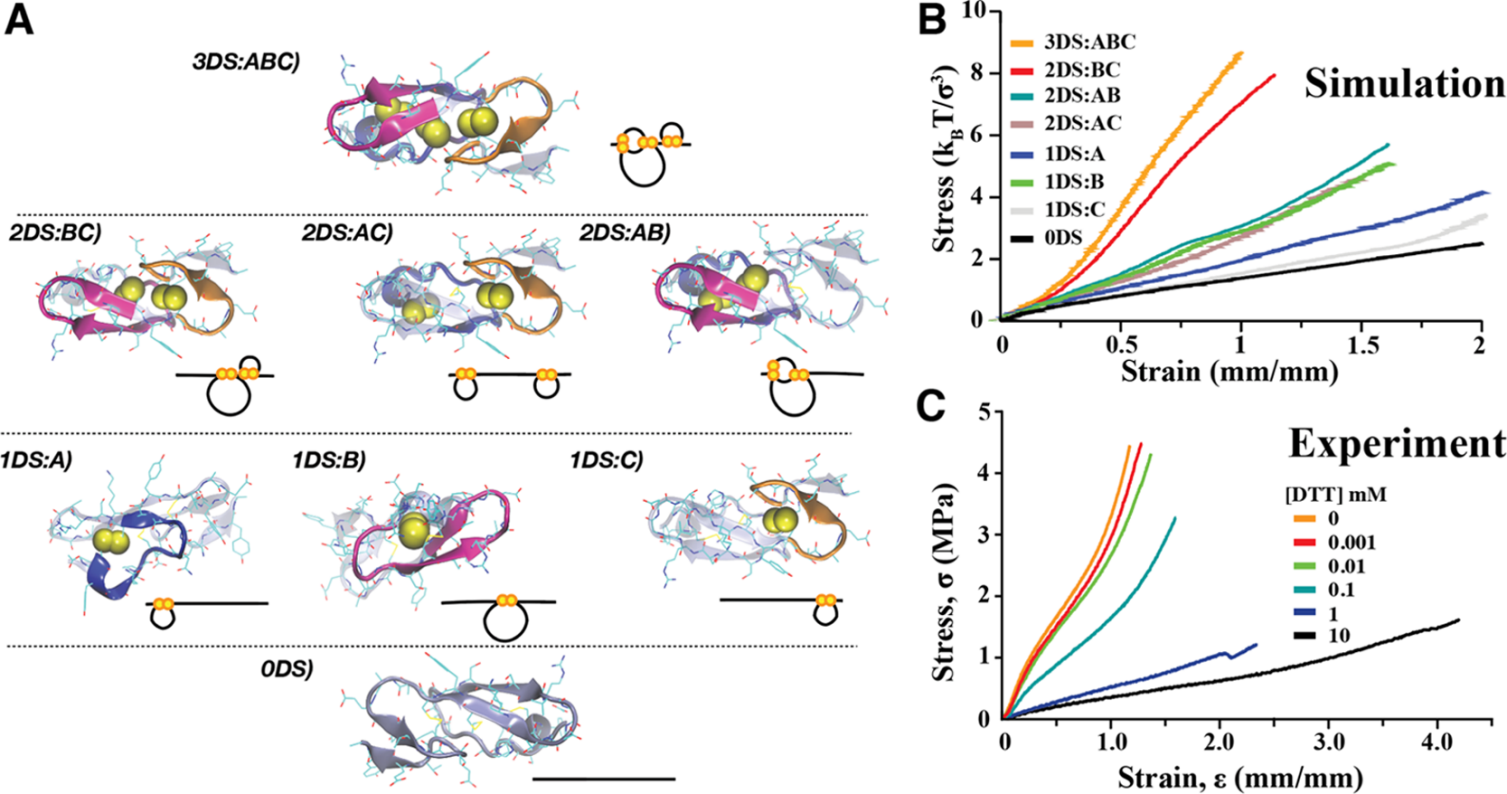
Mechanical effects of disulfide reduction. (A) Model (above) and cartoon representations (below) of each possible disulfide bond combination for an EGF domain, labeled with the number and identity (as defined in Figure 4B) of each disulfide bond present. As an example, the intact EGF domain is 3DS:ABC. (B) CG-MD simulations of the stress–strain response for 29 repeat EGF-like domains. (C) Experimental stress–strain curves for threads treated with increasing amounts of the reducing agent ([DTT] = 0–10 mM).

To understand the mechanical effects of disulfide reduction, we modeled each possible combination of disulfide (DS) bond reduction (Figure 7A) and used CG-MD simulation to model the stress–strain response of 29 repeat EGF-like motifs (Figure 4B). The strongest response occurred when all DS bonds of each EGF domains are intact (Figure 7B, orange, 3DS:ABC). When only one DS bond was broken, there were disparate outcomes. When only DS bond A was broken, there was a slight reduction in the mechanical properties (red [2DS:BC]). However, when either DS bonds B or C were broken, there was a significant decrease in modulus and increase in maximum strain (Figure 7B, brown [2DS:AC] and teal [2DS:AB]). The highest modulus when two DS bonds were broken occurs for the case 1DS-B (green). The modulus was further reduced, and maximum strain increased for the case of 1DS-A (blue) and 1DS-C (gray). The lowest modulus was observed when all DS bonds are reduced (black, 0DS).

To study the experimental effect of DS reduction, thread mechanics were tested after treatment with a reducing agent, DTT (Figure 7C). Low concentrations (0.001–0.01 mM) of DTT generated little effect on the mechanical behavior compared to native threads (Figure 7C, red, orange, and green curves). However, at higher concentrations of 0.1 mM (teal), 1 mM (blue), and 10 mM (black), both the modulus and stress at failure were significantly decreased, whereas the strain at failure significantly increased. Threads treated with 0.1, 1, and 10 mM DTT failed at approximate strains of 150, 230, and 400%, respectively, *vs* native threads which typically fail at strains of 120%.

## Discussion

Although a number of studies have been performed on the ECM of octopus tissues,^42−45^ very few studies have focused on the ECM of octopus egg cases. The tethering egg threads of *O. bimaculoides* were found to be dominated by a single protein named *octovafibrin*. Based on amino acid analysis, acid hydrolysis, and protein electrophoresis, at least 80% of the organic material in the egg threads is *octovafibrin*.

With 29 repeat EGF-like motifs, *octovafibrin* enjoys a high degree of intramolecular crosslinking and joins the ranks of EGF-rich load-bearing ECM proteins. It is tempting to compare octovafibrin sequence to fibrillin, but the lack of a “beads-on-a-string” structure in our TEM (Figure 3D) and the absence of both calcium binding EGF-like and transforming growth factor binding (TB) domains (Figure 4) makes such comparisons tenuous. Fibropellins, proteins that form the apical lamina layer in sea urchin embryos, offer a better comparison. Both *octovafibrin* and fibropellin are present in the reproductive systems of marine invertebrates. Fibropellins also contain up to 21 EGF-like domains that are flanked by an N-terminal C1S-like^46^ domain and a C-terminal avidin-like domain.^47^ The flanking domains contribute to fibropellin assembly/oligomerization, thereby providing a fibrous network while the repeat EGF domains likely mediate a variety of protein–protein interactions.^46^ This suggests that *octovafibrin* assembles analogously with the lectin binding domains driving protein assembly.

Selectins, which also have terminal lectin binding domains preceded by an EGF-like domain, may also provide some insight into the design of *octovafibrin*.^48^ In selectins, the lectin binding domains facilitate adhesive interactions between proteins.^49^ This comparison also suggests that catch bonds may contribute to the mechanical behavior of *octovafibrin*. Catch bonds have the property that their bond strength is increased when mechanical force is applied,^50^ which creates a compelling situation in which *octovafibrin* align end-to-end in a way that enables interactions that strengthen as the thread is strained.

TEM images (Figure 3) showing that *octovafibrin* assembles to form curved fibrillar structures are morphologically similar to previous results obtained with the related incirrata octopus, *Octopus vulgaris*.^51^ We infer from fibril curvature that *octovafibrin* has significant flexibility, and this is consistent with reports that solenoid type proteins with tandem repeats including EGF undergo supercoiling that ranges from circular to bowed to linear.^49,52^ These fibrous stacks also indicate that the proteins may assemble with higher length scale ordering that has been investigated and simulated in ankyrin.^29^

The mechanical behavior of octopus threads also reflects internal ordering as the strain to failure and viscoelastic response resemble those in semicrystalline elastomers.^53−56^ We hypothesize that the opacity of pristine threads results from higher length-scale semicrystalline structures, where light is scattered at the boundaries between the stacked regions (blue squares) and amorphous/void regions (red circles), which is also observed in the TEM images (Figure 3).

We propose the following optical changes: as a pristine thread is loaded, the ordered supercoiled regions are disrupted and the fibrous bundles and the fibers that compose them align, as captured by the changes in SEM (Figure 3B,C), but the threads remain opaque because they likely undergo strain-induced crystallization. Upon unloading, however, the fibers relax to an amorphous state that results in transparent threads. During the second loading cycle, however, the supercoiling is not recovered, thus, the optical contrast increases with strain again due to strain-induced crystallization.

The WAXS data also help us understand what is happening mechanically to the EGF domains in this system. Although EGF domains are present in several load-bearing ECM proteins, very little has been done to investigate strain-induced changes in secondary structure, and *octovafibrin*’s assembly into large monolithic fibers provides an excellent model to explore structure changes. We attribute the WAXS features at *q* = 1.367 Å^–1^ (*d* = 4.6 Å) and *q* = 0.653 Å^–1^ (*d* = 9.6 Å) to β-sheets^57^ present in EGF domains.^19^ The source of the feature at *q* = 0.445 Å^–1^ is unclear, but we suggest that it arises from the EGF domain itself as the *d* spacing (*d* = 14.1 Å) is close to the parameters previously measured for crystalline EGF domains.^58^ Two behaviors are evident by comparing WAXS of unstrained and strained threads. First, the azimuthal profile of the feature at *q* = 0.445 Å^–1^ confirmed that the proteins reoriented under strain so that the EGF domains are packed in a tight, lateral fashion. Second, we found that straining the threads reduced the relative intensity of the β-sheet features. This result is similar to a previous Raman study on fibrillin that indicated strain reduces the prevalence of folds and turns.^59^ Our modeling efforts confirmed this behavior, indicating that the minor β-sheet is readily unfolded because it is not constrained by DS bonds (Figure 7B). The dramatic reduction of the *q* = 1.367 Å^–1^ indicates that the major β-sheet may also unfold, which would require the breaking of at least one DS bond. Indeed, when all the disulfide bonds were reduced, the entire EGF domain (major and minor β-sheets) readily unfolded (Supporting Figure S8).

**Figure 8:**
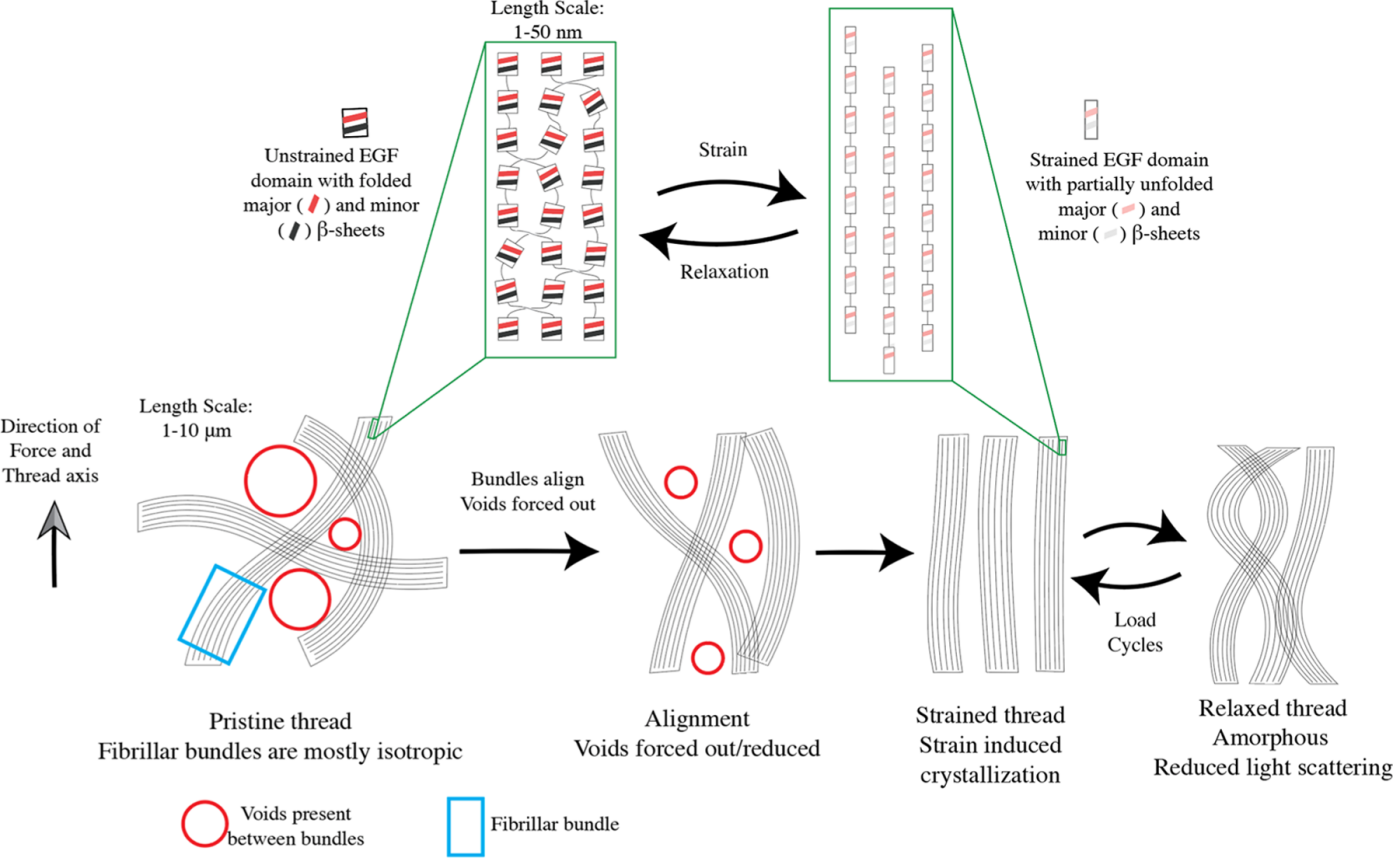
Cartoon model depicting what occurs in the thread during strain. Initially, the thread has domains made of isotropic fibrillar bundles (blue square) with voids interspersed (red circles), which is consistent with what is observed in TEM (Figure 3D). Each component fiber in the bundle is composed of *octovafibrin* proteins (top panel). Prior to deformation, the major and minor β-sheets of EGF-like domains (red and black stripes, respectively) are intact. Upon straining the fibers to align themselves with the axis of tension in addition to the disruption of EGF-like domains (unfolded major and minor β-sheets are indicated with light red and black stripes, respectively). Upon relaxation, the fibers relax to an amorphous state and the EGF-like domains refold.

Taken together, we propose the following model to explain the mechanical behavior of octopus threads (Figure 8). Pristine threads are constructed of randomly oriented fibrillar bundles in a sterically constrained state. For simplicity, our cartoon only shows two dimensions. The fibrils that construct these bundles involve an assembly of multiple *octovafibrin* molecules. When loaded in tension, the bundles align with the axis of tension, breaking the interactions between the bundles, and disrupting the light scattering voids. In addition to the alignment, the EGF-like domains are also strained which causes their β-sheets to unfold. Once unloaded, the fibers relax to an amorphous, and therefore translucent, state rather than the initial sterically constrained state. The response in subsequent stress–strain cycling is then primarily limited to the strain-induced alignment as well as the deformation of the EGF domains.

The lower modulus and reduced hysteresis (Figure 4 and Table S2) of the second loading cycle are then a result of broken interactions between bundled domains as well as increased alignment of the proteins with the axis of tension. This is also reflected in the progressive loss of modulus when repeatedly loaded to higher strain values (Supporting Figure S6). This behavior is observed in semicrystalline elastomers.^53^

The observed cyclic softening resembles Mullin’s effect (*i.e.*, the stress–strain curve shows a softening that depends on the maximum load previously encountered) in polymeric materials but differs in that the threads retain a considerable portion of their mechanical properties after the first cycle. The microstructural reorientation indicated by optical changes in cyclic loading could result from shape memory effects, but further experiments with temperature control would be necessary to confirm this. We further note that our interpretation is likely incomplete as it does not consider complicating viscoelastic effects (Supporting Figure S6C).

In light of the fact that intramolecular bonds can generate topologically complex motifs, yielding mechanically sturdy proteins^60^ and improved bulk mechanical properties,^3^ we sought to analyze the effect of DS bond reduction on thread properties. Interestingly, we observed three regimes in both experimental and simulated analyses. The simulation with all the three EGF bonds (orange) resulted in a stress–strain curve remarkably like that of the native octopus egg threads. The fact that breaking DS-A (the bond between the first and third cysteine) (Figure 7D, purple) indicated that it contributes the least, mechanically speaking. This may be because the remaining DS bonds stabilize both ends of the major β-sheet and at that the portion of sequence constrained by DS-A has some overlap with the sequence constrained by DS-B (between the second and fourth cysteine). However, if either of the DS-B (brown) or DS-C (teal) bonds were broken, one end of the major β-sheet was destabilized, allowing it to unfold, resulting in a greater extension due to more hidden length being exposed.

When DS-B was the lone intact bond (green), it yielded the highest modulus because it provided the largest constraint to the major β-sheet. When only either DS-A (blue) or the DS-C (gray) remains, there was a further decrease in modulus and almost full extension, similar to the response when all DS bonds are broken (black). When fully reduced, the threads exhibited a low modulus and high extensibility, whose behavior typical of uncrosslinked elastomers. In this case, we suggest that the response is entirely due to β-sheet unfolding and untangling of protein molecules. Notably, reduced threads regain their original mechanics following equilibration into DTT-free water (data not shown), suggesting that they readily refold their stable EGF domains. It is unclear if DS bonds on different EGF-like domains are equally prone to DTT reduction. Further study is needed to determine if the various combinations of DS bonds of the thread feature different susceptibilities to reduction and the degree to which disulfides in the C-type lectins contribute. Investigations into how DTT affects the optical properties are of further interest but were not performed here due to the difficulty of acquiring samples.

The present study’s modeling shows that, once elongated, the repeat EGF-like domains result in a high-persistence length polymer. This may be partially responsible for the increased modulus as persistence in length is proportional to Young’s modulus.^61^ The possibility of intermolecular DS bonds that would also increase the modulus cannot be excluded. However, it is reasonable to assume that the EGF-like domains are folded properly, meaning intermolecular DS bond randomization would be uncommon.

Egg thread mechanics conceivably play an important role in the life history of the octopus. Once the eggs are laid, the mother octopus tirelessly broods her eggs for several months until they hatch, whereupon she expires. She constantly moves about her confined den preening and flushing the developing eggs with jets of water. The mechanical properties of egg threads and cases provide protection against predator assault, drag and lift by turbulent/laminar flow, grooming motions of the mother, and various actions by pathogens^62^ and must persist during a long brooding period. Although we focused on egg threads in this study for experimental simplicity, both the egg case and threads appear to be formed during a single continuous process and are biochemically seamless.

## Conclusions

We have studied the biochemical, ultrastructural, and mechanical properties of the reproductive egg threads of *O. bimaculoides*. Threads appear to be made of a single protein, *octovafibrin*, that has 29 repeat EGF-like domains flanked on either side by lectin binding domains. The proteins assemble into semi-ordered fibrous bundles. Threads exhibit a response like semicrystalline elastomers. In cycled tension, softening is observed due to the breaking of interactions between bundles, but there is also a hardening effect resulting from strain-induced alignment. The presence of EGF-like domains, however, adds a fascinating extra dimension to this model. They are capable of dissipating energy *via* domain denaturation when they deform at high strains.

This study confirms that intramolecular crosslinks significantly improve the bulk mechanical properties of polymeric systems. In this case, the removal of intramolecular crosslinks results in greatly reduced modulus but far greater extension. The DS bond between the first and third cysteine of EGF appears to contribute the most to the mechanical response of these domains.

The impact of this study is two-fold: it informs on the rational design of intramolecularly crosslinked systems for increased energy dissipation. Second, it stokes further studies to help define the mechanical contribution of EGF-like domains in other protein systems such as fibrillin and fibropellin.

## Supporting Information Available

Coarse grain modeling of octovafibrin molecules and the MD simulation/thread stretching protocol; extraction and molecular analysis of octovafibrin purified from native threads, as well as supplemental mechanical tests of threads; X-ray analysis of threads; and RMSD simulation of octovafibrin molecules (PDF)
