# Supplementary material for: Mechanical Behavior of Octopus Egg Tethers Composed of Topologically Constrained, Tandemly Repeated EGF Domains
Source: Biomacromolecules. 2023 Jun 9;24(7):3032–42. doi: 10.1021/acs.biomac.3c00088 (PMC10336842; doi:10.1021/acs.biomac.3c00088)
Supplement: Supplementary file 1 — bm3c00088_si_001.pdf [file bm3c00088_si_001.pdf]

Mechanical Behavior of Octopus Egg Tethers Composed of Topologically Constrained, Tandemly Repeated EGF-Domains

William R. Wonderly[1+], Daniel G. DeMartini[2+], Saeed Najafi[1,3+], Marcela Areyano[4], Joan-Emma Shea[1,5], J. Herbert Waite[1,2*]

[1]Department of Chemistry & Biochemistry, University of California Santa Barbara, California, 93106, USA

[2]Department of Molecular, Cell, and Developmental Biology, University of California Santa Barbara, California, 93106, USA

[3]Materials Research Laboratory, University of California Santa Barbara, California 93106, USA

[4]Department of Mechanical Engineering, University of California, Berkeley, California 94720, USA

[5]Department of Physics, University of California Santa Barbara, California 93106, USA

+ Contributed equally

* Corresponding author

SUPPLEMENTARY INFORMATION

Modeling details

We provide a minimalistic representation of the Octopus bimaculoides thread structure and topology by employing an elastic-tube model with a memory of the thread native state. Our coarse-grained (CG) model is based on the spatial organization of the sole C_α atoms, that are connected to the first adjacent amino acids along the polypeptide chain by means of stiff covalent bonds. The only non-bonded interaction to which the monomers are subject is a short-ranged excluded volume, enforcing steric hindrance and preventing the chain from self-crossing. The elastic-tube model of the polypeptide is then provided with bending and torsion potentials, whose reference angles are parametrized based on the thread native state. The latter is constructed by linking 29 aligned identical EGF units, which is extracted from neurogenic locus notch homolog protein crystal structure, see Fig 1. The reference angles that encode the thread native state represent the sole input parameters introduced in the forcefield. In our CG model, the strength of the bending and torsion potentials are uniform and equal.

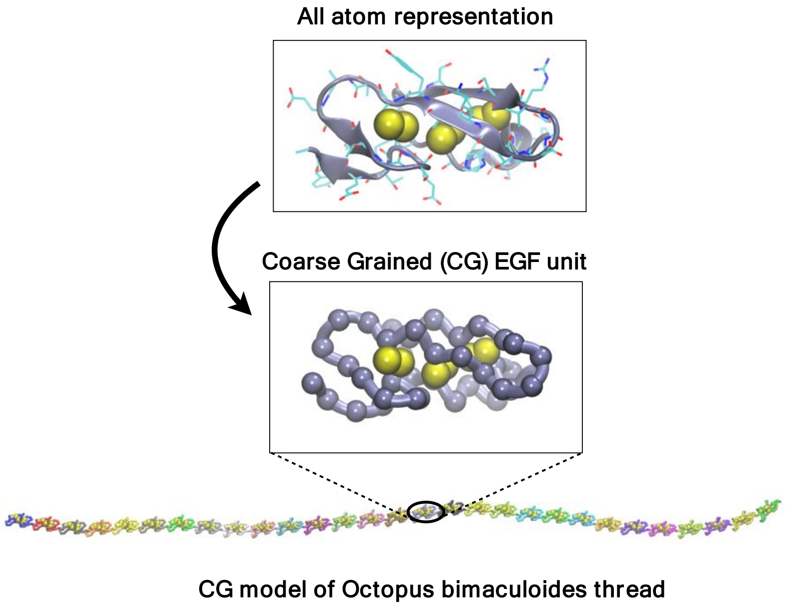

Fig S1. In the top panel, the atomistic model and carton representation of the EGF domain with three topological constraints (the disulfide bonds colored in yellow) is shown. The middle panel represents the elastic-tube coarse-grained (CG) model of the EGF domain with 37 monomers that each represent the C_α atom of the corresponding amino acid. In the bottom panel, 29 identical CG EGF domains are linked to construct the native state of the Octopus bimaculoides thread.

I) Coarse-grained model

The polypeptide of the thread is described as a collection of identical monomers, each representing an amino acid and centered on its C_α atom, connected to its adjacent monomers along the backbone by FENE bonds [1,2]. The monomers exclude each other through a repulsive Weeks-Chandler-Anderson (WCA) potential [3]. Triplets and quadruplets of subsequent beads interact via bending and torsion potentials, respectively. The total potential energy of the system is given by $H = U_{WCA} + U_{FENE} + U_{bend} + U_{tor} + U_{dis}$. The WCA potential is given by:

$$U_{WCA} = \frac{1}{2} \sum_{(i,j),\, i \neq j}^{N} V(d_{i,j})$$

$$V(r) = \begin{cases} 4\epsilon\left[\left(\frac{\sigma}{r}\right)^{12} - \left(\frac{\sigma}{r}\right)^{6} + \frac{1}{4}\right] & if \quad r < 2^{1/6}\sigma \\ 0 & otherwise \end{cases}$$

The FENE potential reads:

$$U_{FENE} = -\sum_{i=0}^{N-2} \frac{\kappa_{fene}}{2}\left(\frac{R_0}{\sigma}\right)^2 ln\left[1 - \left(\frac{d_{i,i+1}}{R_0}\right)^2\right]$$

where $d_{i,i+1} = |\vec{r}_i - \vec{r}_{i+1}|$ is the distance between the *i* and *i + 1* monomers, $R_0 = 1.5\sigma$ is the maximum bond length and $\kappa_{fene} = 30\epsilon$ is the FENE interaction strength; σ and ϵ are units of length and energy, respectively.

The bending and torsion potentials are:

$$U_{bend} = \sum_{i=1}^{N-2} \kappa_{bend}\left(\theta_i(t) - \theta_i^0\right)^2$$

$$U_{tor} = \sum_{i=1}^{N-3} \kappa_{tor}\left(\cos\left(\phi_i(t) - \phi_i^0\right) + \frac{1}{3}\cos\left(3(\phi_i(t) - \phi_i^0)\right)\right)$$

θ_i^0 and ϕ_i^0 are the bending and torsion angles of the *ith* monomer in the native state, respectively. The κ_{bend} and κ_{tor} are the bending and torsion stiffness, here we set $\kappa_{bend} = \kappa_{tor} = 50\epsilon$.

The *ith* and *jth* monomers in disulfide bond, interact through $U_{dis} = \kappa_{dis} \exp\left(\frac{-\left(d_{i,j} - d_{i,j}^0\right)^2}{2o^2}\right)$, where $d_{i,j}^0$ is the equilibrium disulfide bond length between the *ith* and *jth* monomers in native state and $\kappa_{dis} = -100\epsilon$.

II) The MD simulation and thread stretching protocol

The H potential is employed to perform overdamped Molecular Dynamics (MD) simulations in implicit solvent by means of the following Langevin equations of motion:

$$-\frac{\partial H_i}{\partial \vec{r}(t)} - m\gamma\vec{v}_i(t) + \vec{R}_i(t) = 0$$

where m, $\vec{v}_i$, H_i, γ, $\vec{R}_i$ and $\vec{r}$ are the mass, velocity, local potential energy, friction coefficient, random force and coordinate of the *ith* monomer, respectively. The equations of motion of the system are integrated with a first order algorithm at constant temperature with $k_B T = \epsilon$ and $\tau = \sigma \sqrt{m/\epsilon}$ MD time units, and $\Delta t = 0.001\tau$ time step is applied [4]. To investigate the thread response to the external tension along the Z−axis, we perform independent CG-MD simulations. The thread with the uniform elasticity is first equilibrated in the native state where the ends are fixed without clamping. The thread in native structure has a pseudo straight conformation along the Z-axis. We fix one end of the thread and stretch the other end to a certain distance along the Z-axis. We ensure that the disulfide bonds do not break during the stretching by employing a strong disulfide bond strength. For each thread, we ran 20 independent simulations to characterize and quantify the average stress as a function of the strain.

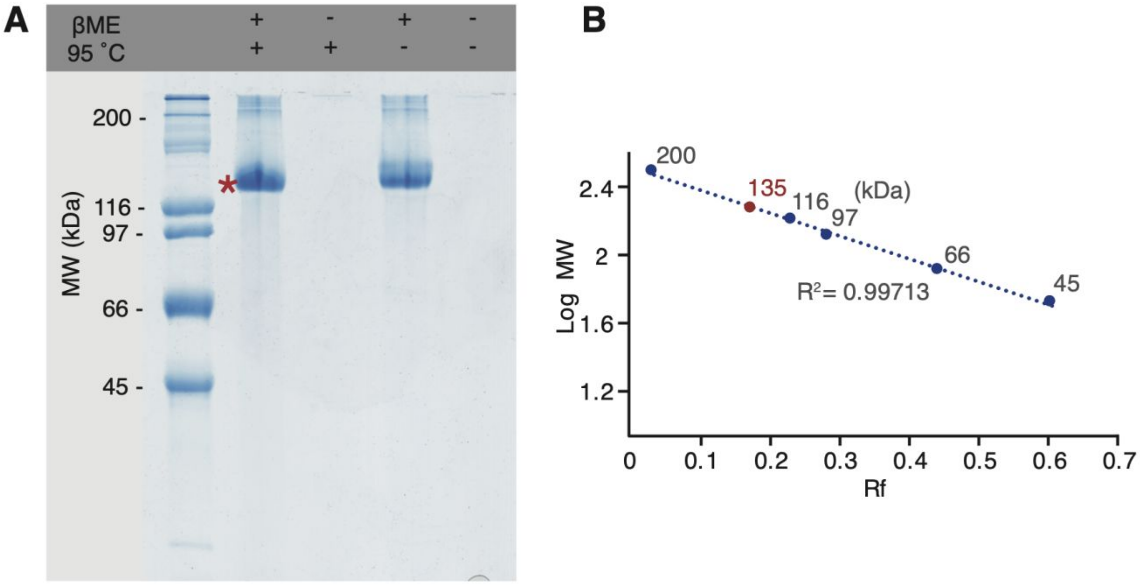

Figure S2. Identification and molecular weight determination of octovafibrin. (a) SDS-PAGE illustrating the effect of β-ME ad heat have on the extraction of octovafibrin. (b) Plot of log(MW) vs relative migration distance (Rf) used to determine the MW of octovafibrin.

Table S1. LC/MS/MS sequences of tryptic *octovafibrin* peptides

m/z detected	z	Sequence
895.052	3	ESKPYICEICAQGFFGDMCQSR
719.3269	2	VDGSHLYSSCANK
981.4711	2	KVHSHFEWVSCGTPYK
654.3021	3	ANGYICDCIEEYLGKR
746.8271	2	CNLGTCPEIDADK
649.2854	2	FGCLDGWTGER
460.2007	2	DSVGCEPR
1101.53	4	CCSLKGNVVWLESSFELNYVATHLLDGMASQTDTWVGAK
472.2005	2	SYEGAMNR
663.8494	4	HVKNNLWYSEHPKPSSNVAFIGK
794.8041	2	NDGTCQDMEESFR
858.3525	2	CDCKPGYCGPLCTK
799.3462	2	FTCVCDSGYYGIR
597.2805	3	NGGICIIDGYEYKCK
428.8793	3	CKCPKPYFGK
436.8781	3	CTCKPAYVGPR
506.192	2	HYECSCR
593.2744	2	QGLSFVCSCK
872.6804	3	CRPGYLGTDCSTFDYCSDGPCK
542.759	2	NSGTCYVVGK
830.915	2	KGSVYTCICQIGFK
440.6815	2	FTCHCR

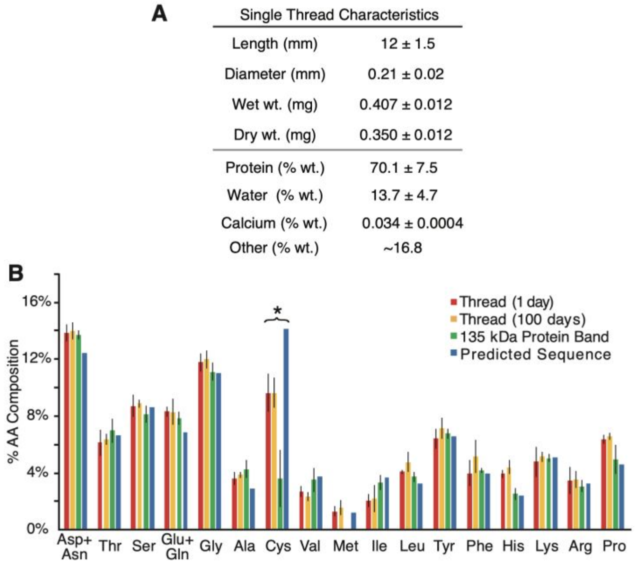

Figure S3. Characteristics of octopus threads. (a) Table displaying average thread characteristics. (b) Chart comparing the amino acid analysis results of whole threads at 1 and 100 days, purified 135 kDa protein, and the predicted protein sequence.

Table S2. *Average toughness and hysteresis values of octopus threads.*

Cycle #	Toughness (MPa)	Hysteresis (%)
1	2.48 ± 0.48	52.5 ± 1.1
2	2.12 ± 0.45	48.2 ± 1.8
3	2.04 ± 0.49	47.0 ± 1.0
8	1.99 ± 0.43	46.7 ± 2.0

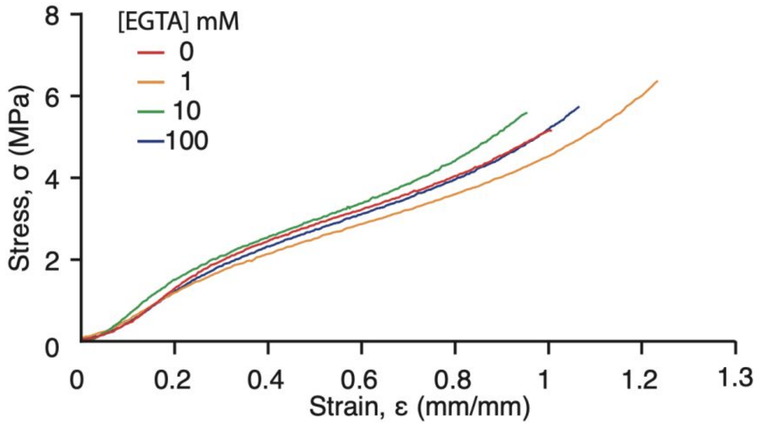

Figure S4. Effects of a chelating agent (EGTA) on the mechanical properties of threads.

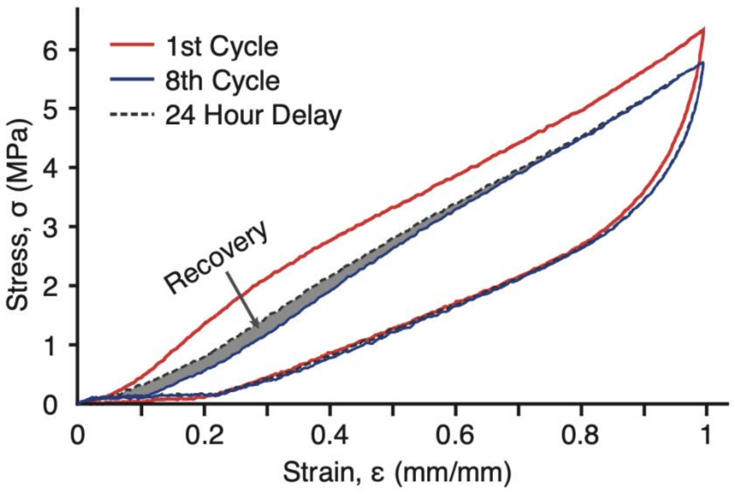

Figure S5. Cyclic loading behavior of pristine thread (red), the profile of the 8th load cycle (blue), and the profile of a load cycle after allowing 24 hours of recovery (dashed line). The shaded area indicates the recovery.

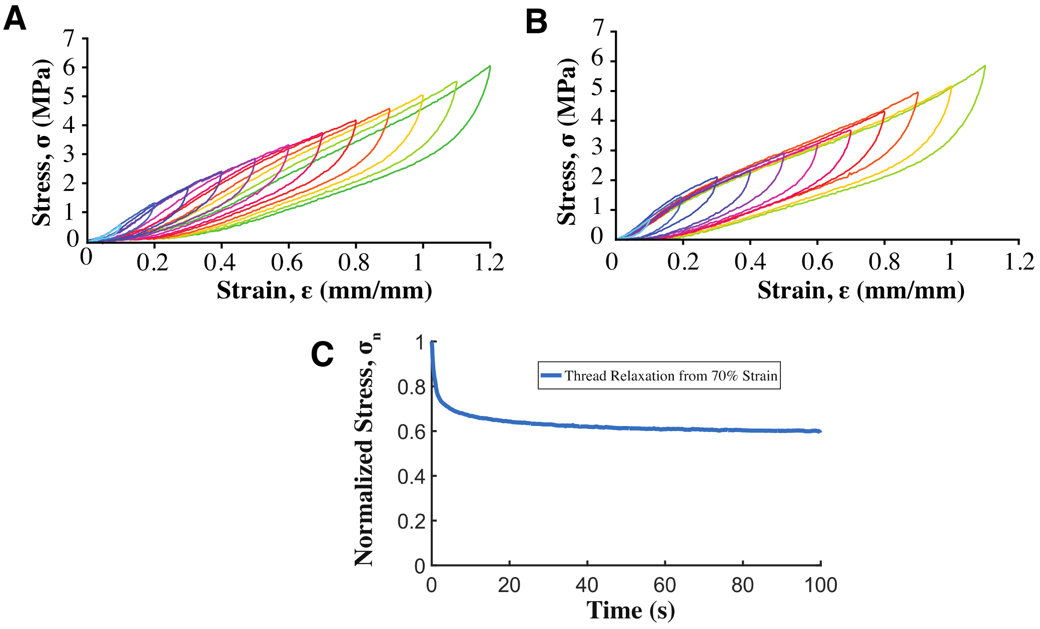

Figure S6. Mechanical behavior of octopus threads. (a) Stress-strain behavior of a single thread subjected to repeated loading cycles going to increasing strain values. (b) Stress-strain behavior of pristine threads under cyclic loading to increasing strain values. (c) Stress relaxation behavior of a thread held at 70% strain.

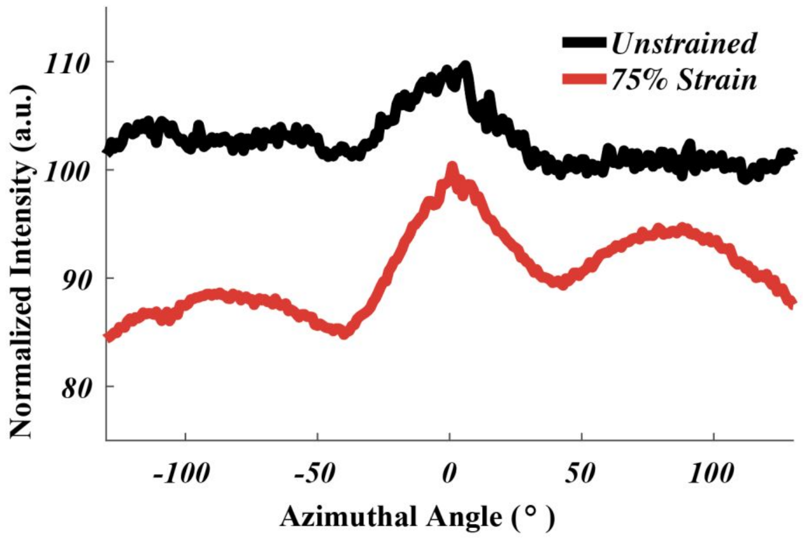

Figure S7. The azimuthal profile generated by the shaded blue box in Fig. 6C corresponding to the peak at q = 0.445Å^{-1}. The data for unstrained threads are shown in black and the strained threads, which show increased alignment, is shown in red.

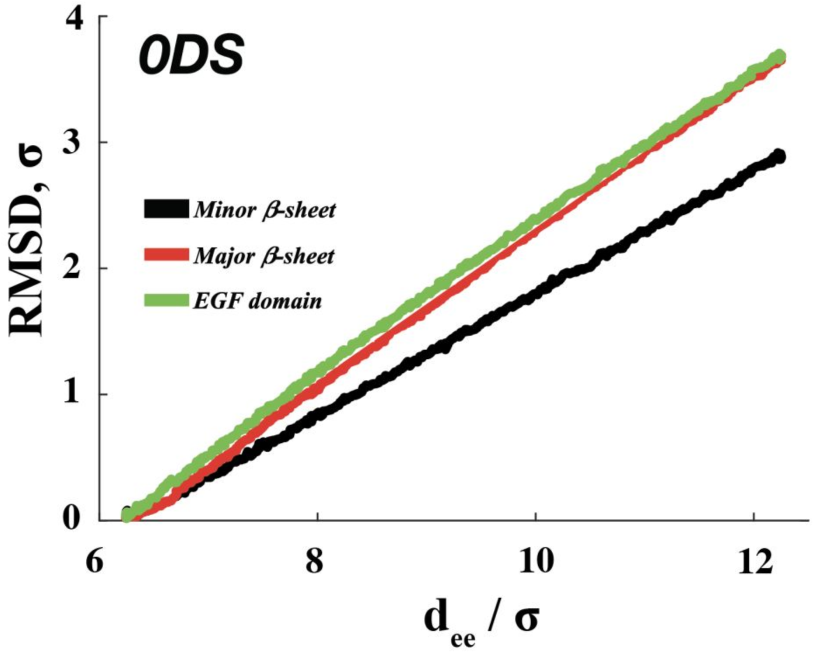

Figure S8. RMSD simulation of a strained EGF-like domain whose disulfide bonds have been completely reduced that shows unfolding of the minor β-sheet (black), major β-sheet (red), and full EGF domain (green).

References

[1] G. S. Grest and K. Kremer, Phys. Rev. A 33, 3628 (1986).
[2] K. Kremer and G. S. Grest, The Journal of Chemical Physics 92, 5057 (1990).
[3] J. D. Weeks, D. Chandler, and H. C. Andersen, The Journal of Chemical Physics 54, 5237 (1971).
[4] D. C. Rapaport, The Art of Molecular Dynamics Simulation, 2nd ed. (Cambridge University Press, 2004).
